# Midwives’, Obstetricians’, and Recently Delivered Mothers’ Perceptions of Remote Monitoring for Prenatal Care: Retrospective Survey

**DOI:** 10.2196/10887

**Published:** 2019-04-15

**Authors:** Dorien Lanssens, Thijs Vandenberk, Joy Lodewijckx, Tessa Peeters, Valerie Storms, Inge M Thijs, Lars Grieten, Wilfried Gyselaers

**Affiliations:** 1 Limburg Clinical Research Program Mobile Health Unit Hasselt University Diepenbeek Belgium; 2 Faculty of Medicine and Life Sciences Hasselt University Diepenbeek Belgium; 3 Department of Cardiology & Future Health Ziekenhuis Oost-Limburg Genk Belgium

**Keywords:** remote monitoring, gestational hypertensive diseases, questionnaires, monitoring, ambulatory, hypertension, pregnancy-induced, surveys and questionnaires

## Abstract

**Background:**

The Pregnancy Remote Monitoring (PREMOM) study enrolled pregnant women at increased risk of developing hypertensive disorders of pregnancy and investigated the effect of remote monitoring in addition to their prenatal follow-up.

**Objective:**

The objective of this study was to investigate the perceptions and experiences of remote monitoring among mothers, midwives, and obstetricians who participated in the PREMOM study.

**Methods:**

We developed specific questionnaires for the mothers, midwives, and obstetricians addressing 5 domains: (1) prior knowledge and experience of remote monitoring, (2) reactions to abnormal values, (3) privacy, (4) quality and patient safety, and (5) financial aspects. We also questioned the health care providers about which issues they considered important when implementing remote monitoring. We used a 5-point Likert scale to provide objective scores. It was possible to add free-text feedback at every question.

**Results:**

A total of 91 participants completed the questionnaires. The mothers, midwives, and obstetricians reported positive experiences and perceptions of remote monitoring, although most of them had no or little prior experience with this technology. They supported a further rollout of remote monitoring in Belgium. Nearly three-quarters of the mothers (34/47, 72%) did not report any problems with taking the measurements at the required times. Almost half of the mothers (19/47, 40%) wanted to be contacted within 3 to 12 hours after abnormal measurement values, preferably by telephone.

**Conclusions:**

Although most of midwives and obstetricians had no or very little experience with remote monitoring before enrolling in the PREMOM study, they reported, based on their one-year experience, that remote monitoring was an important component in the follow-up of high-risk pregnancies and would recommend it to their colleagues and pregnant patients.

**Trial Registration:**

ClinicalTrials.gov NCT03246737; https://clinicaltrials.gov/ct2/show/NCT03246737 (Archived by WebCite at http://www.webcitation.org/76KVnHSYY)

## Introduction

### Background

Due to demographic changes and rapid improvements in medical technology, the health care sector is confronted with major challenges and great opportunities. The care and follow-up of a pregnant woman and her unborn baby is an important element in health care. Due to the changing lifestyles of pregnant women, the number of high-risk pregnancies has risen over the last few decades [1-3]. Therefore, there is a need to increase the efficiency of follow-up for these pregnancies without loss of quality of care. Telemedicine presents an opportunity for the follow-up of high-risk pregnancies.

Defined as the use of information and communication technologies for supporting health and health-related activities [4], telemedicine is not simply an addition to conventional care, but rather is implemented in current private and public health care approaches. Remote monitoring (RM) is a type of telemedicine that has a broad definition. It is useful for conducting medical practice from a distance and has been used in a wide variety of electronic health care applications [5]. RM can be performed either by live monitoring of vital parameters or asynchronously, whereby data obtained in the patient’s home environment are sent to the health care provider [4]. Examples of chronic diseases that could benefit from RM include diabetes, heart failure, and cardiac arrhythmias [6-8].

### The Pregnancy Remote Monitoring Study

The Pregnancy Remote Monitoring (PREMOM) study, which started in January 2015 in a tertiary center, Ziekenhuis Oost-Limburg (Genk, Belgium), involved RM of pregnant women at high risk of hypertensive disorders of pregnancy (HDP). The PREMOM study design, data collection method, and first promising results are described in detail elsewhere [9,10] (NCT03246737). Briefly, the PREMOM study was performed in the outpatient clinic of a second-level prenatal center where pregnant women with HDP received RM or conventional care. Women in the RM group received obstetric surveillance using a blood pressure monitor, an activity tracker, and a weight scale. They were asked to measure blood pressure twice a day, measure their weight once a week, and wear an activity tracker for 24 hours/day. These data were automatically sent by Wi-Fi or Bluetooth to an online platform, which was developed by the Mobile Health Unit (University of Hasselt, Hasselt, Belgium). A midwife reviewed the data every workday. The activity data were tracked to investigate the influence of daily activity (eg, total number of steps per day) on the development of HDP. Predetermined thresholds (systolic blood pressure >140 mm Hg, diastolic blood pressure >90 mm Hg, or weight gain >1 kg/day) were configured and resulted in automatically generated alarm signals.

The midwife discussed the alarm events with the obstetrician in charge to discuss the appropriate medical treatment. The midwife contacted the patients to give additional instructions about possible medical interventions such as altered medication regimens. These therapeutic interventions were according to local management.

### Objectives

Because, to our knowledge, the perceptions or expectations of a prenatal RM follow-up program have not previously been investigated, we performed a quantitative survey of recently delivered women and health care providers (ie, both the obstetricians and the midwives). Here, we describe the main outcomes, which cover the following domains: (1) prior knowledge and experience of RM, (2) reactions to abnormal values, (3) privacy, (4) quality and patient safety, and (5) financial aspects. We also asked health care providers about important aspects to consider when implementing RM.

## Methods

### Questionnaires

The research group of the Mobile Health Unit designed 3 questionnaires: (1) for women who were followed up with RM during their last pregnancy, (2) for midwives working at the Ziekenhuis Oost-Limburg (Genk, Belgium) who were involved in the use of RM, and (3) for consulting obstetricians working at several hospitals in Limburg. The questionnaires assessed the 5 domains to elucidate PREMOM participants’ perceptions and experiences of RM and were based on the 6 building blocks established by the Mobile Health Working Group of the Voka Health Community (Brussels, Belgium): (1) protection of data, privacy, and the use of big data; (2) national and international regulations and responsibility; (3) quality, accessibility, and patient safety; (4) technology and interoperability; (5) financial aspects and business models; and (6) supportive policy frameworks in telemedicine. Here we discuss the results of the descriptive PREMOM questionnaires on the domains prior knowledge and experience of RM, reactions to abnormal values, privacy, quality and patient safety, and financial aspects, which are important to health care providers for further implementation of RM. We drafted the questionnaires in April 2016 using Survey Monkey 2016 (SurveyMonkey Inc) for completion online. We assessed all questions using 5-point Likert scales to obtain objective scores. It was possible to add free-text feedback at every question.

### Participants

We sent the questionnaires in April 2016 to the women, midwives, and obstetricians who participated in the PREMOM study in 2015. We excluded student midwives and doctors in training.

### Data Collection

The study participants received an email from the research team with a link to the online survey. We sent email reminders to all participants at 9 and 23 days after the first invitation.

### Analysis

We assessed mean scores and ranks for each question using descriptive analytical methods. The number of participants included in the analyses of individual questions was different from the total number of analyzed questionnaires because some mothers, midwives, and obstetricians did not complete all of the questions. We required at least half of the questionnaire to be completed for inclusion in the analysis. We conducted statistical analysis with IBM SPSS version 24.0 (IBM Corporation).

### Ethical Considerations

We sent a generic link, to maintain anonymity, to the participants to fill in the survey. A bulk email was sent with the participants’ email addresses included in the BCC field to ensure that there were no recognizable personal elements in the email.

The email was addressed to “Dear Madam” or “Dear Colleague” to remove the personal salutation to participate in this study. In addition, no personal participant identification number was requested or electronically reported when completing the questionnaires. Unique internet protocol addresses prevented duplicate responses to the questionnaires. The Medical Ethics Committee of Ziekenhuis Oost-Limburg approved this study (no. 14/078U).

## Results

### Participant Characteristics

The study population consisted of 158 people: 92 mothers (58%), 52 midwives (33%), and 14 obstetricians (9%). The total number of pregnant women involved in the PREMOM study was 119, so we contacted 77% (92/119) of the participants after their delivery. The 27 women who did not participate didn’t answer their phone, didn’t have an email address, or didn’t speak Dutch. We excluded 1 obstetrician from the final analyses for completing less than 50% of the questionnaire. Therefore, the total response rate was 57% (91/158). Multimedia Appendix 1 shows the questionnaire and response summaries for midwives, Multimedia Appendix 2 shows the questionnaire and response summaries for obstetricians, and Multimedia Appendix 3 shows the questionnaire and response summaries for patients in English translation from the original Dutch. Table 1 shows the participants’ demographic characteristics.

### Prior Knowledge and Experience of Remote Monitoring

**Figure 1:**
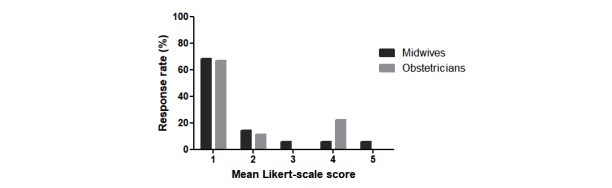
Summary of responses from the midwives and obstetricians on the question “Please indicate with a score from 1 (strongly disagree) to 5 (strongly agree): I had already experience with RM before this study.” RM: remote monitoring.

The first part of the questionnaire examined the midwives’ and obstetricians’ prior knowledge or experience of RM. Overall, 29 of the 35 midwives (83%) and 7 of the 9 (78%) obstetricians reported little or no experience of RM (Figure 1).

The midwives were also asked about their experience of RM as a threat to their daily work. The majority (29/35, 83%) of midwives did not perceive RM as a threat to their work.

### Timing and Method of Communication in Case of an Event

Nearly three-quarters (34/47, 72%) of the participating mothers reported that they had no problems with taking the measurements at the requested times. Of the 7 mothers (15%) who reported difficulties with the recommended measurements, 4 (57%) were between 36 and 40 years old, 2 (29%) were between 26 and 30 years old, and 1 (14%) was between 31 and 35 years old.

We also asked participants about the acceptable time limit for being contacted by their health care provider in case of an unexpected event. Of the 47 women who completed the questionnaire, 13 (28%) preferred to be contacted within 3 hours of the event, 19 (40%) preferred to be contacted between 3 and 12 hours, and 15 (32%) preferred to be contacted more than 12 hours after the event (Table 2).

**Table 1:** Characteristics of participants.

Study group			Responses, n (%)
**Women who were remotely monitored during their last pregnancy (n=47)**			
	**Age (years)**		
		<20	0 (0)
		20-25	5 (11)
		26-30	16 (34)
		31-35	21 (4)
		36-40	4 (9)
		>40	1 (2)
	**Primigravidity**		
		Primipara	21 (45)
		Multipara	26 (55)
	**History of hypertensive disorders of pregnancy**		
		Yes	17 (36)
		No	10 (21)
		N/A^a^	20 (4)
	**Level of education**		
		Lower secondary school	4 (9)
		Higher secondary school	12 (26)
		High school	20 (43)
		University	11 (23)
**Midwives (n=35)**			
	**Age (years)**		
		20-25	3 (9)
		26-30	8 (23)
		31-35	7 (20)
		36-40	3 (9)
		>40	14 (40)
	**Time in practice (years)**		
		<5	4 (11)
		5-15	15 (43)
		16-25	8 (23)
		>25	8 (23)
	**Main activity on nursing unit**		
		Delivery unit	11 (31)
		Maternity	8 (23)
		Maternal intensive care	10 (29)
		Prenatal visits	6 (17)
**Obstetricians (n=9)**			
	**Time in practice (years)**		
		<5	1 (11)
		5-15	6 (67)
		16-25	0 (0)
		>25	2 (22)
	**Main activity in their specialty**		
		Delivery unit	4 (44)
		Obstetrics	4 (44)
		Oncology	1 (11)

^a^N/A: not available.

**Table 2:** Summary of responses to the question “Within how much time do you want to be contacted about events?” (n=47).

Response categories	Responses, n (%)
<3 hours	13 (28)
3-12 hours	19 (40)
12-24 hours	7 (15)
24-48 hours	5 (11)
>48 hours	3 (6)

Interestingly, 4 of the 5 mothers (80%) aged less than 25 years asked to be contacted within 3 hours of an event. We also asked the participants how to contact them following an event. The participants’ first preference was to be contacted by telephone (weighted average 4.55/5), their second preference was during a prenatal consultation (weighted average 3.94/5), and the third preference was to be contacted by text messages (weighted average 3.17/5). Finally, we asked the participants who should contact the women in case of an event. The mothers and midwives stated that the obstetrician should be the first to contact the pregnant woman after an abnormal event. However, the obstetricians reported that their representing researcher should be the first health care provider to contact the pregnant woman in case of an event.

### Privacy

We asked the mothers if they felt that regularly sharing their health data was a threat to their privacy. Most (41/47, 87%) of the mothers reported that they did not have any negative concerns about privacy, while 3 mothers (aged 36-40 years) reported that sharing health data posed a threat to their privacy.

### Quality and Patient Safety

**Figure 2:**
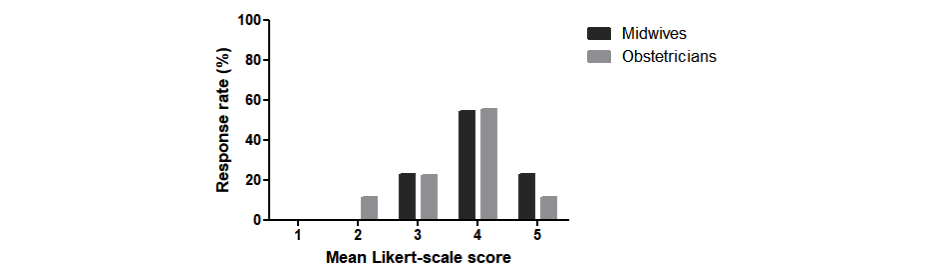
Summary of responses from the midwives and obstetricians to the question “Do you believe that RM improves the care for pregnant women with an increased risk of gestational complications? Please indicate with a score from 1 (strongly disagree) to 5 (strongly agree).” RM: remote monitoring.

We asked the mothers about the importance of RM in the follow-up of their pregnancy. Most (42/47, 89%) of the mothers had a positive response to this question. Meanwhile, 28 of the 35 (80%) midwives reported that RM provided added value to pregnant women, and 27 of the 35 (77%) midwives felt that RM improved the care for high-risk pregnancies. This percentage is slightly higher than that of the 9 obstetricians, 6 (67%) of whom felt that RM provided added value to their patients (Figure 2).

Moreover, 8 of the 9 (89%) obstetricians responded, based on their experience of the PREMOM study, that the pregnant women did not request additional prenatal consultations for the purpose of viewing their own vital parameters. Finally, 39 of the 47 (83%) mothers reported that RM gave them a feeling of safety.

### Financial Aspect

An important element in new health care practices is their financial cost. Therefore, the relative and absolute costs of each component in telemonitoring programs need to be evaluated. All 3 groups of participants reported that the cost of RM should be as low as possible, and about half of the mothers expected RM to be free, without a personal contribution from the patient (25/47, 53%). It is also important to obtain information on any potential payer of RM. The mothers expected the hospital to be the main payer, followed by their health insurance provider, whereas midwives and obstetricians felt that the pregnant women should also personally contribute to the cost of RM.

### Further Implementation of Remote Monitoring

We asked the midwives and obstetricians about important factors to support the implementation of RM into daily practice. Most of the midwives (31/35, 89%) felt that it is important to receive additional training on “the information that must be given to pregnant women about hypertensive disorders of pregnancy and the added value of remote monitoring for this disease,” as one of the midwives noted. Obstetricians (7/9, 78%) considered this 11 percentage points less necessary than did midwives. The obstetricians (8/9, 89%) felt that training on the technical handling of the devices (eg, installation and common problems) was the most important factor. About three-quarters of midwives (27/35, 77%) had the same response to this question. For the final evaluation of the project, we asked the obstetricians whether they would recommend RM to pregnant women and their colleagues. Overall, 6 of the 9 (67%) obstetricians supported this service and would recommend it to their patients, while 7 of the 9 (78%) obstetricians would recommend RM to their colleagues. Finally, 6 of the 9 (67%) obstetricians recommended that this follow-up should be expanded to all pregnant women in Belgium who are at increased risk of HDP.

## Discussion

### Principal Findings

RM is a relatively new field in obstetric research. Earlier studies of telemedicine that included cervical dilation and preterm labor as the main outcome demonstrated that transmitting uterine activity by telecommunication resulted in significantly prolonged pregnancy survivals [11,12]. Studies of telemedicine for patients with gestational diabetes mellitus demonstrated lower levels of frustration and concerns about their diabetes and a better acceptance of their diabetic condition [13], greater feelings of self-efficacy [14], and reduced unscheduled face-to-face visits [15,16] in the telemedicine group compared with the control group. Reduced costs [17,18] and greater feelings of maternal satisfaction [14,19,20] were obtained when telemedicine was used in obstetric care. Newborns had a higher gestational age at delivery [18] and were less likely to have a low birth weight [11,18] or to be admitted to the neonatal intensive care unit [11,18] in the telemedicine group compared with a control group. Fetuses with abnormal versus normal fetal heart rate at home monitoring were more likely to have a lower gestational age [21]. Recent studies about RM in women at risk for HDP demonstrated that they had fewer inductions, more spontaneous labors, and fewer maternal and neonatal hospitalizations when compared with conventional care [9,10]. Also, providing women at risk for HDP with RM was shown to be cost-effective for the health care system [22].

To our knowledge, this is the first quantitative survey of an RM program for prenatal care. The results show that most midwives and obstetricians had no or very little experience of RM before they participated in the PREMOM study. After taking part in the PREMOM study and the survey, the midwives reported that RM is not a threat to their daily work. Most of the mothers who were supervised by RM during their last pregnancy did not experience any problems with taking the required measurements at the specified times. Most of the mothers thought that it would be acceptable to be contacted within 3 to 12 hours after an abnormal value, and they preferred to be contacted by telephone.

The study of Giardina et al [23] showed the duality of feedback after a normal or an abnormal test. Nearly two-thirds of clinicians agreed that patients should receive direct feedback after a normal test. However, most physicians in the study expressed concerns about direct notification of clinically abnormal test results based on a patient’s anxiety, confusion, lack of expertise to interpret the results, and seeking unreliable information to understand the results, and concerns that the patient would seek care without consulting their provider. The results of that study showed that doctors would be comfortable with a time interval of 24 to 48 hours for contacting a patient after an abnormal test result [23].

Privacy is a critical aspect of health care and RM [24]. The mothers in our study did not have concerns about sharing their health data with their obstetrician. As mentioned by Piwek and Ellis [25], data security and patients’ privacy are essential elements for the adoption of digital smartphone research methods. Some risk-averse participants might be unwilling to share their clinical data with a commercial partner. However, none of the participants reported any privacy breaches using RM during this study.

The quality of care experienced by pregnant women with increased risk of HDP was enhanced by RM, as reported by the surveyed mothers and health care providers, and supported by the results of the prior pilot study [9]. Mothers who were involved in the project reported that RM gave them a feeling of security throughout their pregnancy. Previous research concluded that pregnant women with gestational diabetes mellitus had an increased sense of self-regulation when they used RM to send their blood glucose levels to their midwives [14,16]. Other research showed that pregnant women had heightened feelings of maternal satisfaction when using RM as additional care with their labor induction [19,20].

The mothers, midwives, and obstetricians included in this study reported that RM is an important aspect of the follow-up of high-risk pregnancies. An issue that raises important questions in telemedicine is the rather low adherence rate to RM, especially during long-term monitoring [26-29].

Measuring blood pressure, body weight, and activity every day is a prerequisite to ensure adequate monitoring of pregnant women, although this may appear burdensome to many of them. However, the mothers surveyed in this study did not experience this obstacle.

The obstetricians stated that they would recommend RM to colleagues and other pregnant women. Most of the obstetricians proposed extending RM to all women with high-risk pregnancies in Belgium. The obstetricians and midwives also reported that all users need additional training to support the implementation of RM. Earlier research already mentioned the challenge in training these obstetricians and midwives in the collection and interpretation of results, as well as incorporation of the remote patient data into routine clinical practice [30].

### Strengths and Limitations of the Study

Despite the increased implementation of RM in health care, its use is still limited in obstetrics. To our knowledge, this was the first study to investigate obstetricians’, midwives’, and recently delivered mothers’ perceptions of the use of RM for preterm follow-up of pregnancies at risk for HDP. Another strength of this study is that it included stakeholders involved in the use of RM, including health care providers and actual users. The questionnaires also allowed the participants to explain their responses to each question, allowing us to obtain supplementary information. Furthermore, the participants could complete the questionnaire anonymously. Finally, a relatively high percentage of participants in the PREMOM study completed the questionnaires.

Although the results of this study are encouraging, there are several limitations that should be considered for future research. First, because the questionnaire was completed anonymously, it was not possible to contact the individual participants to request additional information. Second, the questionnaire was digital and completed in an uncontrolled condition, so it is unclear whether the participants were exposed to external influences when they completed the questionnaire. Additionally, the 3 groups in this study had small sample sizes, which could affect external validity. Third, this study was performed in a local hospital, which can reduce the generalization of the results. Fourth, the study included obstetricians who worked at several hospitals in Limburg, but the midwives and mothers were enrolled from only a single center (Ziekenhuis Oost-Limburg).

### Recommendations for Further Research

Both the mothers and the midwives felt that the obstetrician should be responsible for contacting the patient after an abnormal event, while the obstetricians suggested that their reporting researcher should be responsible for this task. This may relate to the organization of prenatal care in Belgium, where midwives act nearly as obstetric nurses rather than independently, and the prenatal care for pregnant women mostly is performed by an obstetrician, whether a pregnant woman has a high- or a low-risk pregnancy. It is remarkable that none of these 3 groups felt that this could be a task of the patient’s midwife, although the researcher (DL) in this study is certified as a midwife. Still, the allocation of the responsibility for RM coordination to the midwives seems logical, as they act as an intermediary between the pregnant woman and the obstetrician. Clearly, further research is needed to understand the factors underlying this opinion and how it could be changed.

Additionally, both the mothers and the health care workers stated that RM should be offered for free or that they wanted to pay as little as possible for the RM services. Although we have conducted a cost-effectiveness study, which showed that RM makes saving costs possible for the health care system [22], we have not yet studied the willingness to pay. This study would have an additional value to set a price for RM services when the health care provider or the hospital requests it.

Further, although 67% of the obstetricians would recommend RM to their patients and 78% would recommend it to their colleagues, the obstetricians who would not recommend it did not give any reason for this. A follow-up qualitative questionnaire to investigate the underlying reasons for this should be helpful for the further implementation of RM in standard prenatal care for women at risk for HDP.

Interestingly, the mothers preferred to be contacted between 3 and 12 hours after an abnormal clinical measurement. This implies that the clinical data should be monitored 24 hours a day, 7 days a week in order to evaluate and interpret the vital parameters of pregnant women and permit an intervention if necessary. Therefore, we recommend developing a system of care aimed at providing these services. As our previous studies showed, our RM prenatal follow-up would result in the prenatal ward having a lower burden of treating women with HDP [9,10]. Finally, although we invited the mothers with abnormal events to additional prenatal consultations to assess fetal and maternal well-being, none of the patients or the participating obstetricians believed that this was needed and as such did not threaten to overload the health care system. These findings may contradict the statement that the medicalization of childbirth has gone too far and that too many medical interventions are performed in pregnancies, which has arisen from a variety of sources [31-36].

### Conclusions

Although most midwives and obstetricians had no or very little experience with RM before they participated in the PREMOM study, they felt that it is an important aspect of the follow-up of pregnancies at risk for HDP. Most of the mothers who were supervised by RM during their last pregnancy thought that it was acceptable to be contacted within 3 to 12 hours after an abnormal value, and they preferred to be contacted by telephone. Most women had no concerns about regularly sharing their clinical data with their obstetrician, and they reported that RM gave them a feeling of security throughout their pregnancy. To our knowledge, this is the first quantitative survey of mothers, midwives, and obstetricians involved in an RM program in prenatal care. Further studies are needed to understand the underlying opinions of mothers, midwives, and obstetricians regarding RM. Based on our findings, we propose developing a care system with 24-hours-a-day, 7-days-a-week surveillance by RM of mothers at high risk of HDP.

## Appendix

Multimedia Appendix 1 Questionnaire for midwives, with responses.

Multimedia Appendix 2 Questionnaire for obstetricians, with responses.

Multimedia Appendix 3 Questionnaire for patients, with responses.

## Abbreviations

HDP: hypertensive disorders of pregnancy
PREMOM: Pregnancy Remote Monitoring
RM: remote monitoring
