# Supplementary material for: Midwives’, Obstetricians’, and Recently Delivered Mothers’ Perceptions of Remote Monitoring for Prenatal Care: Retrospective Survey
Source: J Med Internet Res. 2019 Apr 15;21(4):e10887. doi: 10.2196/10887 (PMC6487343; doi:10.2196/10887)
Supplement: Multimedia Appendix 1 [file jmir_v21i4e10887_app1.pdf]

Questionnaire for midwives

1. Background information

Variable	N	Response frequencies				
**How old are you?**						
		**20 – 25 years**	**26 – 30 years**	**31 – 35 years**	**36 – 40 years**	**> 40 years**
	35	3 (8.57%)	8 (22.86%)	7 (20.00%)	3 (8.57%)	14 (40.00%)
**Which is your highest level of education?**						
		**Student**	**High school**	**University**		
	35	0 (0.00%)	35 (100.00%)	0 (0.00%)		
**How many years of work experience do you have?**						
		**< 5 years**	**5 – 15 years**	**16 – 25 years**	**> 25 years**	
	35	8 (22.86%)	15 (42.86%)	4 (11.43%)	8 (22.86%)	
**Before you started this job, did you already have work experiences in …**						
		**A private practice**	**A secondary care center**	**A primary care center**		
Yes	35	2 (5.71%)	12 (34.29%)	17 (50.00%)		
**On which department are you working at this moment?**						
		**Delivery room**	**Maternity**	**Prenatal ward**	**Prenatal consultations**	
	35	11 (31.43%)	8 (22.86%)	10 (28.57%)	6 (17.14%)	

2. Perception about remote monitoring

Variable	N	Response frequencies				
**On a scale from 1 to 5 (with 5 being mostly agree) how would you rate the use of remote monitoring as being an added value for …**						
		**1**	**2**	**3**	**4**	**5**
The pregnant woman	35	0 (0.00%)	2 (5.71%)	5 (14.29%)	18 (51.43%)	10 (28.57%)
The midwife	35	0 (0.00%)	2 (5.71%)	9 (25.71%)	15 (42.86%)	9 (25.71%)
The general practitioner	35	1 (2.86%)	4 (11.43%)	8 (22.86%)	14 (40.00%)	8 (22.86%)
The obstetrician	35	0 (0.00%)	1 (2.86%)	4 (11.43%)	16 (45.71%)	14 (40.00%)
The society	35	0 (0.00%)	1 (2.86%)	14 (40.00%)	11 (31.43%)	9 (25.71%)
**On a scale from 1 to 5 (with 5 being mostly agree), how would you rate the devices which are used on this moment to detect pre-eclampsia?**						
		**1**	**2**	**3**	**4**	**5**
The blood pressure monitor	35	0 (0.00%)	0 (0.00%)	7 (20.00%)	16 (45.71%)	12 (34.29%)
The activity tracker	35	2 (5.71%)	3 (8.57%)	17 (48.57%)	12 (34.29%)	1 (2.86%)
The weight scale	35	1 (2.86%)	2 (5.71%)	11 (31.43%)	16 (45.71%)	5 (14.29%)
**Rate the following questions on a scale from 1 to 5 (with 5 being mostly agree).**						
		**1**	**2**	**3**	**4**	**5**
Before the study started, I did already know what remote monitoring meant.	35	8 (22.86%)	12 (34.29%)	7 (20.00%)	6 (17.14%)	2 (5.71%)
Before the study started, I did already know what remote monitoring aims.	35	8 (22.86%)	11 (31.43%)	8 (22.86%)	7 (20.00%)	1 (2.86%)
Before the study started, I already had some practical experiences with remote monitoring.	35	24 (68.57%)	5 (14.29%)	2 (5.71%)	2 (5.71%)	2 (5.71%)
The use of remote monitoring is an added value on my daily work package.	35	13 (37.14%)	9 (25.71%)	6 (17.14%)	5 (14.29%)	2 (5.71%)
The use of remote monitoring is an threat for my daily work package.	35	24 (68.57%)	5 (14.29%)	4 (11.43%)	2 (5.71%)	0 (0.00%)

3. Education

Variable	N	Response frequencies				
**On a scale from 1 to 5 (with 5 being mostly agree), on which item do you think (para)medical personnel should have an extra education before they start to work with remote monitoring?**						
		**1**	**2**	**3**	**4**	**5**
Which explanation we have to give the patients before they start to use remote monitoring.	35	0 (0.00%)	0 (0.00%)	4 (11.43%)	16 (45.71%)	15 (42.86%)
How we can support the patients in order to have a high compliance rate.	35	0 (0.00%)	0 (0.00%)	8 (22.86%)	14 (40.00%)	13 (37.14%)
About the technology of the devices which are used for remote monitoring.	35	0 (0.00%)	1 (2.86%)	7 (20.00%)	13 (37.14%)	14 (40.00%)
About the protocols which have to be used in the remote monitoring of different complications.	35	0 (0.00%)	0 (0.00%)	5 (14.29%)	15 (42.86%)	15 (42.86%)

4. Quality and patient safety

Variable	N	Response frequencies				
**Rate the following questions on a scale from 1 to 5 (with 5 being mostly agree).**						
		**1**	**2**	**3**	**4**	**5**
Do you think that remote monitoring will make the patient unnecessarily worried?	35	5 (14.29%)	18 (51.43%)	10 (28.57%)	2 (5.71%)	0 (0.00%)
Do you think that remote monitoring will give the patient a false safety feeling?	35	6 (17.14%)	13 (37.14%)	11 (31.43%)	5 (14.29%)	0 (0.00%)
Do you think that remote monitoring will make your work easier?	35	4 (11.43%)	5 (14.29%)	17 (48.57%)	9 (25.71%)	0 (0.00%)
Do you think that a remote monitoring prenatal follow-up program the care for women with a high risk for gestational hypertensive disorders improves?	35	0 (0.00%)	0 (0.00%)	8 (22.86%)	19 (54.29%)	8 (22.86%)
Do you think that a shift in healthcare is possible by using remote monitoring?	35	1 (2.86%)	1 (2.86%)	7 (20.00%)	19 (54.29%)	7 (20.00%)
Do you think that remote monitoring will be a standard tool in healthcare in the future?	35	0 (0.00%)	0 (0.00%)	8 (22.86%)	19 (54.29%)	8 (22.86%)
Do you think it would be agreeable when more remote monitoring studies will be performed in the future?	35	1 (2.86%)	0 (0.00%)	16 (45.71%)	14 (40.00%)	4 (11.43%)
Do you think that the blood pressure of the pregnant women can be elevated because of the stress for the result?	35	2 (5.71%)	4 (11.43%)	9 (25.71%)	19 (54.29%)	1 (2.86%)
Do you think that the result of remote monitoring nog represent the reality?	35	2 (5.71%)	11 (31.43%)	17 (48.57%)	5 (14.29%)	0 (0.00%)
**On a scale from 1 to 5 (with 5 being mostly agree), do you think the measurements will be done correctly by the pregnant women?**						
		**1**	**2**	**3**	**4**	**5**
The bloodpressure	35	0 (0.00%)	2 (5.71%)	13 (37.14%)	17 (48.57%)	3 (8.57%)
The weight	35	0 (0.00%)	2 (5.71%)	14 (40.00%)	15 (42.86%)	4 (11.43%)
The activity- and sleeppatern	35	0 (0.00%)	1 (2.86%)	16 (45.71%)	15 (42.86%)	3 (8.57%)
**Rate the following questions on a scale from 1 to 5 (with 5 being mostly agree).**						
		**1**	**2**	**3**	**4**	**5**
Patients will be reassured by the positive results on the app and will be less likely to have (unnecessary) prenatal visits.	35	0 (0.00%)	2 (5.71%)	16 (45.71%)	16 (45.71%)	1 (2.86%)
Patient will be more worried because they can see their measurements and will have more unscheduled prenatal visits.	35	2 (5.71%)	8 (22.86%)	18 (51.43%)	6 (17.14%)	1 (2.86%)
Patients need to have an extra prenatal consult when deviations are noticeable in the results.	35	2 (5.71%)	1 (2.86%)	11 (31.43%)	18 (51.43%)	3 (8.57%)
The feedback of the results to the patients is essential.	35	0 (0.00%)	0 (0.00%)	10 (28.57%)	16 (45.71%)	9 (25.71%)
A lot of information will be become on a way which doesn't take much time	35	0 (0.00%)	2 (5.71%)	7 (20.00%)	19 (54.29%)	7 (20.00%)
I don't like the additional reports I get from the researches.	35	5 (14.29%)	8 (22.86%)	21 (60.00%)	1 (2.86%)	0 (0.00%)
A researcher is a new member of the multidisciplinary team. The communication gets more complicated in that way.	35	3 (8.57%)	10 (28.57%)	17 (48.57%)	3 (8.57%)	2 (5.71%)

5. Technology & interoperability

Variable	N	Response frequencies				
**Rate the following questions on a scale from 1 to 5 (with 5 being mostly agree).**						
		**1**	**2**	**3**	**4**	**5**
Do you think it would be an added value when the results of the remote motoring will be automatically send to the patients' medical files?	35	1 (2.86%)	0 (0.00%)	2 (5.71%)	21 (60.00%)	11 (31.43%)

6. Feedback

Variable	N	Response frequencies					
**Indicate which answer is applicable**							
		**Daily**	**Weekly**	**Monthly**	**Only when the values are abnormal**	**At its own request**	**Never**
When do you think the pregnant woman needs to receive an overview of her measurement?	35	6 (17.14%)	15 (42.86%)	5 (14.29%)	6 (17.14%)	3 (8.57%)	0 (0.00%)
When do you think the pregnant woman needs to receive an overview of the deliberation between the caregivers (based on her measurements) about her results?	35	0 (0.00%)	18 (51.43%)	6 (17.14%)	9 (25.71%)	2 (5.71%)	0 (0.00%)
**When do you think the pregnant woman needs to receive an overview (without a consultation by the midwife or the obstetrician) of her measurement on the following ways?**							
		**Daily**	**Weekly**	**Monthly**	**Only when the values are abnormal**	**At its own request**	**Never**
By telephone	35	0 (0.00%)	11 (31.43%)	3 (8.57%)	15 (42.86%)	7 (20.00%)	8 (22.86%)
By email	35	2 (5.71%)	12 (34.29%)	6 (17.14%)	4 (11.43%)	10 (28.57%)	6 (17.14%)
By text message	35	2 (5.71%)	5 (14.29%)	1 (2.86%)	2 (5.71%)	11 (31.43%)	16 (45.71%)
At the prenatal consultation	35	0 (0.00%)	10 (28.57%)	12 (34.29%)	10 (28.57%)	9 (25.71%)	1 (2.86%)
**When do you think the pregnant woman needs to receive a consultation of her measurement on the following ways?**							
		**Daily**	**Weekly**	**Monthly**	**Only when the values are abnormal**	**At its own request**	**Never**
By telephone	35	0 (0.00%)	11 (31.43%)	3 (8.57%)	16 (48.71%)	7 (20.00%)	5 (14.29%)
By email	35	3 (8.57%)	12 (34.29%)	6 (17.14%)	3 (8.57%)	9 (25.71%)	7 (20.00%)
By text message	35	2 (5.71%)	4 (11.43%)	2 (5.71%)	3 (8.57%)	10 (28.57%)	16 (45.71%)
At the prenatal consultation	35	1 (2.86%)	12 (34.29%)	12 (34.29%)	13 (37.14%)	6 (17.14%)	1 (2.86%)
**On a scale from 1 to 5 (with 5 being mostly relevant), when do you think the pregnant woman needs to be informed in the following situation?**							
		**1**	**2**	**3**	**4**	**5**	
When the measurements doesn't have any abnormalities	35	7 (20.00%)	10 (28.57%)	11 (31.43%)	2 (5.71%)	5 (14.29%)	
When the measurements can't be overviewed due to technical problems	35	3 (8.57%)	2 (5.71%)	5 (14.29%)	10 (28.57%)	15 (42.86%)	
When an adequate follow-up isn't possible due to insufficient compliance by the patient	35	1 (2.86%)	0 (0.00%)	6 (17.14%)	10 (28.57%)	18 (51.43%)	
When the measurements can't be overviewed due to problems by the online dashboard	35	1 (2.86%)	1 (2.86%)	8 (22.86%)	10 (28.57%)	15 (42.86%)	
When the privacy of the pregnant women is threated due to problems with the data security	35	1 (2.86%)	1 (2.86%)	7 (20.00%)	9 (25.71%)	17 (48.57%)	
**Of who do you think the pregnant woman needs to receive an overview or feedback about the results … (Multiple answers are correct)**							

		Autom-atically	The obste-trician	The mid-wife	The general practi-tioner	The resear-cher	No prefer-ence	Other
When there are no abnormal measurements	35	2 (5.71%)	30 (85.71%)	19 (54.29%)	8 (22.86%)	17 (48.57%)	1 (2.86%)	1 (2.86%)
When there are abnormal measurements	35	0 (0.00%)	32 (91.43%)	15 (42.86%)	5 (14.29%)	17 (48.57%)	0 (0.00%)	1 (2.86%)

Do you think that the partner of the pregnant women needs to be involved in the services remote monitoring offers?

		Yes	No
	35	22 (62.86%)	13 (37.14%)

On a scale from 1 to 5 (with 5 being mostly relevant), how would you involve the partner of the pregnant woman in this program?

		1	2	3	4	5
By involving the partner in the intake conversation	35	2 (5.71%)	2 (5.71%)	2 (5.71%)	15 (42.86%)	14 (40.00%)
By giving the partner an overview of the results of the pregnant women on his Smartphone	35	15 (42.86%)	6 (17.14%)	5 (14.28%)	7 (20.00%)	2 (5.71%)
By informing the partner when measurements are missed	35	11 (31.45%)	3 (8.57%)	9 (25.71%)	9 (25.71%)	3 (8.57%)
By giving feedback of the results to the partner	35	15 (42.86%)	1 (2.86%)	7 (20.00%)	8 (22.86%)	4 (11.43%)

7. Privacy

Variable	N	Response frequencies				
**Rate the following questions on a scale from 1 to 5 (with 5 being mostly agree).**						
		**1**	**2**	**3**	**4**	**5**
Would you offer remote monitoring to your patients when their privacy can't guaranteed fully?	35	15 (42.86%)	7 (20.00%)	11 (31.43%)	1 (2.86%)	1 (2.86%)
Do you think that remote monitoring is an threat for the patient data?	35	14 (40.00%)	7 (20.00%)	13 (37.14%)	1 (2.86%)	0 (0.00%)
How import is patient privacy towards the ability to identify gestational complications in an early stage?	35	7 (20.00%)	4 (11.43%)	14 (40.00%)	5 (14.29%)	5 (14.29%)

8. Financial considerations

Variable	N	Response frequencies						
**What is an acceptable price to pay for remote monitoring?**								
		**€10/month**	**€25/month**	**€50/month**	**Other**			
Yes	35	20 (57.14%)	2 (5.71%)	0 (0.00%)	13 (37.14%)			
**Who has to pay for remote monitoring?**								
		**The patient**	**The obste-trician**	**The society**	**The hospital**	**The obste-trician & the society**	**The patient & the society**	**The patient, the obste-trician and the society**
Yes	35	0 (0.00%)	1 (2.86%)	11 (31.43%)	2 (5.71%)	1 (2.86%)	12 (34.29%)	8 (22.86%)
**Do you think a distinction has to be made in a reimbursement by the insurance between patients with a low risk or a high risk?**								
		**Yes**	**No**					
	35	21 (60.00%)	14 (40.00%)					
**On a scale from 1 to 5 (with 5 being mostly relevant), which items are important to implement remote monitoring into daily practice?**								
		**1**	**2**	**3**	**4**	**5**		
An additional training (with an certificate)	35	4 (11.43%)	1 (2.66%)	6 (17.14%)	15 (42.86%)	9 (25.71%)		
Hiring additional staff	35	2 (5.71%)	3 (8.57%)	12 (34.29%)	11 (31.43%)	7 (20.00%)		
When an independent service will follow-up the patients and gives us information about the remote monitoring services	35	2 (5.71%)	3 (8.57%)	11 (31.43%)	12 (34.29%)	7 (20.00%)		
When this is an activity which only happens during the working hours	35	2 (5.71%)	7 (20.00%)	14 (40.00%)	8 (22.86%)	4 (11.43%)		
When additional space will be made into the work planning so no extra workload will be created	35	0 (0.00%)	0 (0.00%)	7 (20.00%)	13 (37.14%)	15 (42.86%)		
