# Supplementary material for: Midwives’, Obstetricians’, and Recently Delivered Mothers’ Perceptions of Remote Monitoring for Prenatal Care: Retrospective Survey
Source: J Med Internet Res. 2019 Apr 15;21(4):e10887. doi: 10.2196/10887 (PMC6487343; doi:10.2196/10887)
Supplement: Multimedia Appendix 2 [file jmir_v21i4e10887_app2.pdf]

Questionnaire for Obstetrician

1. Background information

Variable	N	Response frequencies				
**How many years of work experience do you have in …?**						
		**0**	**< 5 years**	**5 – 15 years**	**16 – 25 years**	**> 25 years**
A private practice	9	3 (33.33%)	1 (11.11%)	4 (44.44%)	0 (0.00%)	1 (11.11%)
A secondary care center	9	7 (77.77%)	1 (11.11%)	1 (11.11%)	0 (0.00%)	0 (0.00%)
A primary care center	9	0 (0.00%)	0 (0.00%)	7 (77.77%)	1 (11.11%)	1 (11.11%)
**How many percent of work experiences do you have on …?**						
		**0 – 20%**	**21 – 40%**	**41 – 60%**	**61 – 80%**	**81 – 100%**
The delivery room	9	2 (22.22%)	2 (22.22%)	2 (22.22%)	3 (33.33%)	0 (0.00%)
Gynaecology	9	3 (33.33%)	6 (66.66%)	0 (0.00%)	0 (0.00%)	0 (0.00%)
Oncology	9	6 (66.66%)	2 (22.22%)	1 (11.11%)	0 (0.00%)	0 (0.00%)
Fertility	9	8 (88.88%)	1 (11.11%)	0 (0.00%)	0 (0.00%)	0 (0.00%)

2. Perception about remote monitoring

Variable	N	Response frequencies				
**On a scale from 1 to 5 (with 5 being mostly agree) how would you rate the use of remote monitoring as being an added value for …**						
		**1**	**2**	**3**	**4**	**5**
The pregnant woman	9	1 (11.11%)	0 (0.00%)	1 (11.11%)	4 (44.44%)	3 (33.33%)
The midwife	9	2 (22.22%)	0 (0.00%)	4 (44.44%)	3 (33.33%)	0 (0.00%)
The general practitioner	9	2 (22.22%)	1 (11.11%)	3 (33.33%)	3 (33.33%)	0 (0.00%)
The gyneacologist	9	1 (1.11%)	0 (0.00%)	1 (11.11%)	6 (66.67%)	1 (11.11%)
The society	9	1 (11.11%)	0 (0.00%)	3 (33.33%)	5 (55.56%)	0 (0.00%)
**On a scale from 1 to 5 (with 5 being mostly agree), how would you rate the devices which are used on this moment to detect pre-eclampsia?**						
		**1**	**2**	**3**	**4**	**5**
The blood pressure monitor	9	0 (0.00%)	1 (11.11%)	0 (0.00%)	5 (55.56%)	3 (33.33%)
The activity tracker	9	2 (22.22%)	4 (44.44%)	3 (33.33%)	0 (0.00%)	0 (0.00%)
The weight scale	9	1 (1.11%)	3 (33.33%)	3 (33.33%)	2 (22.22%)	0 (0.00%)
**Rate the following questions on a scale from 1 to 5 (with 5 being mostly agree).**						
		**1**	**2**	**3**	**4**	**5**
I can easily convince my patients of the added value of remote monitoring	9	1 (11.11%)	0 (0.00%)	1 (11.11%)	3 (33.33%)	4 (44.44%)
The relationship between me and my patients is strongly changed in a positive way	9	1 (11.11%)	1 (11.11%)	5 (55.56%)	2 (22.22%)	0 (0.00%)
I am obligated to change the prenatal follow-up of my patients by using remote monitoring	9	1 (11.11%)	4 (44.44%)	2 (22.22%)	1 (11.11%)	1 (11.11%)

3. Application

Variable	N	Response frequencies				
**Rate the following questions on a scale from 1 to 5 (with 5 being mostly agree).**						
		**1**	**2**	**3**	**4**	**5**
Before the study started, I did already know what remote monitoring meant.	9	1 (11.11%)	3 (33.33%)	1 (11.11%)	2 (22.22%)	2 (22.22%)
Before the study started, I did already know what remote monitoring aims.	9	2 (22.22%)	0 (0.00%)	2 (22.22%)	3 (33.33%)	2 (22.22%)
Before the study started, I already had some practical experiences with remote monitoring.	9	6 (66.67%)	1 (11.11%)	0 (0.00%)	2 (22.22%)	0 (0.00%)
The use of remote monitoring is an added value on my daily work package.	9	0 (0.00%)	1 (11.11%)	2 (22.22%)	5 (55.56%)	1 (11.11%)
The use of remote monitoring is an threat for my daily work package.	9	1 (11.11%)	4 (44.44%)	2 (22.22%)	1 (11.11%)	1 (11.11%)

4. Education

Variable	N	Response frequencies				
**On a scale from 1 to 5 (with 5 being mostly agree), on which item do you think (para)medical personnel should have an extra education before they start to work with remote monitoring?**						
		**1**	**2**	**3**	**4**	**5**
Which explanation we have to give the patients before they start to use remote monitoring.	9	1 (11.11%)	1 (11.11%)	0 (0.00%)	3 (33.33%)	4 (44.44%)
How we can support the patients in order to have a high compliance rate.	9	0 (0.00%)	1 (11.11%)	1 (11.11%)	3 (33.33%)	4 (44.44%)
About the technology of the devices which are used for remote monitoring.	9	0 (0.00%)	1 (11.11%)	0 (0.00%)	3 (33.33%)	5 (55.56%)
About the protocols which have to be used in the remote monitoring of different complications.	9	1 (11.11%)	0 (0.00%)	0 (0.00%)	4 (44.44%)	4 (44.44%)

5. Quality and patient safety

Variable	N	Response frequencies				
**Rate the following questions on a scale from 1 to 5 (with 5 being mostly agree).**						
		**1**	**2**	**3**	**4**	**5**
Do you think that remote monitoring will make the patient unnecessarily worried?	9	3 (33.33%)	3 (33.33%)	2 (22.22%)	1 (11.11%)	0 (0.00%)
Do you think that remote monitoring will give the patient a false safety feeling?	9	1 (11.11%)	5 (55.56%)	2 (22.22%)	1 (11.11%)	0 (0.00%)
Do you think that remote monitoring will make your work easier?	9	1 (11.11%)	0 (0.00%)	5 (55.56%)	3 (33.33%)	0 (0.00%)
Do you think that a remote monitoring prenatal follow-up program the care for women with a high risk for gestational hypertensive disorders improves?	9	0 (0.00%)	1 (11.11%)	2 (22.22%)	5 (55.56%)	1 (11.11%)
Do you think that a shift in healthcare is possible by using remote monitoring?	9	1 (11.11%)	0 (0.00%)	3 (33.33%)	5 (55.56%)	0 (0.00%)
Do you think that remote monitoring will be a standard tool in healthcare in the future?	9	1 (11.11%)	0 (0.00%)	1 (11.11%)	7 (77.78%)	0 (0.00%)
Do you think it would be agreeable when more remote monitoring studies will be performed in the future?	9	0 (0.00%)	1 (1.11%)	1 (11.11%)	7 (77.78%)	0 (0.00%)
Do you think that the blood pressure of the pregnant women can be elevated because of the stress for the result?	9	1 (11.11%)	1 (11.11%)	2 (22.22%)	5 (55.56%)	0 (0.00%)
Do you think that the result of remote monitoring not represent the reality?	9	0 (0.00%)	7 (77.78%)	2 (22.22%)	0 (0.00%)	0 (0.00%)
**On a scale from 1 to 5 (with 5 being mostly agree), do you think the measurements will be done correctly by the pregnant women?**						
		**1**	**2**	**3**	**4**	**5**
The bloodpressure	9	0 (0.00%)	1 (11.11%)	1 (11.11%)	7 (77.78%)	0 (0.00%)
The weight	9	1 (11.11%)	0 (0.00%)	5 (55.56%)	3 (33.33%)	0 (0.00%)
The activity- and sleeppatern	9	0 (0.00%)	1 (11.11%)	5 (55.56%)	3 (33.33%)	0 (0.00%)
**Rate the following questions on a scale from 1 to 5 (with 5 being mostly agree).**						
		**1**	**2**	**3**	**4**	**5**
Patients will be reassured by the positive results on the app and will be less likely to have (unnecessary) prenatal visits.	9	1 (11.11%)	0 (0.00%)	1 (11.11%)	7 (77.78%)	0 (0.00%)
Patients will be more worried because they can see their measurements and will have more unscheduled prenatal visits.	9	0 (0.00%)	8 (88.89%)	1 (11.11%)	0 (0.00%)	0 (0.00%)
Patients need to have an extra prenatal consult when deviations are noticeable in the results.	9	0 (0.00%)	2 (22.22%)	3 (33.33%)	4 (44.44%)	0 (0.00%)
The feedback of the results to the patients is essential.	9	0 (0.00%)	1 (11.11%)	1 (11.11%)	7 (77.78%)	0 (0.00%)
A lot of information will be become on a way which doesn't take much time	9	1 (11.11%)	2 (22.22%)	1 (11.11%)	5 (55.56%)	0 (0.00%)
I don't like the additional reports I get from the researches.	9	1 (11.11%)	6 (66.67%)	1 (11.11%)	0 (0.00%)	1 (11.11%)
A researcher is a new member of the multidisciplinary team. The communication gets more complicated in that way.	9	0 (0.00%)	4 (44.44%)	3 (33.33%)	1 (11.11%)	1 (11.11%)
**On a scale from 1 to 5 (with 5 being mostly agree), which factors can help to become a successful implementation of remote monitoring?**						
		**1**	**2**	**3**	**4**	**5**
Support by the ICT department of the hospital	9	0 (0.00%)	1 (11.11%)	2 (22.22%)	5 (55.56%)	1 (11.11%)
Support by the researchers	9	0	0	1	4	4

		(0.00%)	(0.00%)	(11.11%)	(44.44%)	(44.44%)
Devotion of the gyneacologist	9	0<br>(0.00%)	1<br>(11.11%)	0<br>(0.00%)	6<br>(66.67%)	2<br>(22.22%)
Devotion of the midwife	9	0<br>(0.00%)	1<br>(11.11%)	1<br>(11.11%)	7<br>(77.78%)	0<br>(0.00%)
Devotion of the hospital	9	0<br>(0.00%)	2<br>(22.22%)	0<br>(0.00%)	6<br>(66.67%)	1<br>(11.11%)

6. Aims

Variable	N	Response frequencies				
**Rate the following questions on a scale from 1 to 5 (with 5 being mostly agree).**						
		**1**	**2**	**3**	**4**	**5**
The insights of the patients in their own behavior and (normal) parameters can be enlarged by the use of remote monitoring	9	1 (11.11%)	0 (0.00%)	4 (44.44%)	3 (33.33%)	1 (11.11%)
Patients can make the connection between their lifestyle and abnormal measurements by using remote monitoring	9	1 (11.11%)	3 (33.33%)	2 (22.22%)	3 (33.33%)	0 (0.00%)
Prenatal admissions will less occur and will be of a shorter duration by using remote monitoring.	9	1 (11.11%)	2 (22.22%)	2 (22.22%)	4 (44.44%)	0 (0.00%)
The patient will have a decent reaction on her results	9	0 (0.00%)	3 (33.33%)	6 (66.67%)	0 (0.00%)	0 (0.00%)
The antihypertensive therapy can be adjusted by the measurements which are become by using remote monitoring	9	1 (11.11%)	0 (0.00%)	0 (0.00%)	7 (77.78%)	1 (11.11%)
I can spend more time to the patients who really need this by using remote monitoring	9	0 (0.00%)	2 (22.22%)	1 (11.11%)	6 (66.67%)	0 (0.00%)
Remote monitoring is an added value to the quality of our healthcare	9	0 (0.00%)	1 (11.11%)	1 (11.11%)	7 (77.78%)	0 (0.00%)

7. Technology & interoperability

Variable	N	Response frequencies				
**Rate the following questions on a scale from 1 to 5 (with 5 being mostly agree).**						
		**1**	**2**	**3**	**4**	**5**
Do you think it would be an added value when the results of the remote motoring will be automatically send to the patients' medical files?	9	1 (11.11%)	0 (0.00%)	1 (11.11%)	4 (44.44%)	3 (33.33%)

8. Feedback

Variable	N	Response frequencies					
**Indicate which answer is applicable**							
		**Daily**	**Weekly**	**Monthly**	**Only when the values are abnormal**	**At its own request**	**Never**
When do you think the pregnant woman needs to receive an overview of her measurement?	9	1 (11.11%)	5 (55.56%)	1 (11.11%)	1 (11.11%)	2 (22.22%)	0 (0.00%)
When do you think the pregnant woman needs to receive an overview of the deliberation between the caregivers (based on her measurements) about her results?	9	5 (55.55%)	2 (22.22%)	0 (0.00%)	4 (44.44%)	0 (0.00%)	0 (0.00%)
**When do you think the pregnant woman needs to receive an overview (<u>without</u> a consultation by the midwife or the gyneacologist) of her measurement on the following ways?**							
		**Daily**	**Weekly**	**Monthly**	**Only when the values are abnormal**	**At its own request**	**Never**
By telephone	9	1 (11.11%)	0 (0.00%)	0 (0.00%)	5 (55.56%)	0 (0.00%)	4 (44.44%)
By email	8	2 (25.00%)	2 (25.00%)	0 (0.00%)	2 (25.00%)	0 (0.00%)	2 (25.00%)
By text message	9	2 (22.22%)	1 (11.11%)	0 (0.00%)	2 (22.22%)	2 (22.22%)	4 (44.44%)
At the prenatal consultation	9	4 (44.44%)	0 (0.00%)	0 (0.00%)	2 (22.22%)	1 (11.11%)	3 (33.33%)
**When do you think the pregnant woman needs to receive a consultation of her measurement on the following ways?**							
		**Daily**	**Weekly**	**Monthly**	**Only when the values are abnormal**	**At its own request**	**Never**
By telephone	9	1 (11.11%)	1 (11.11%)	0 (0.00%)	3 (33.33%)	2 (22.22%)	2 (22.22%)
By email	9	0 (0.00%)	2 (22.22%)	0 (0.00%)	1 (11.11%)	1 (11.11%)	5 (55.56%)
By text message	9	0 (0.00%)	2 (22.22%)	0 (0.00%)	1 (11.11%)	1 (11.11%)	5 (55.56%)
At the prenatal consultation	9	7 (77.78%)	1 (11.11%)	0 (0.00%)	2 (22.22%)	1 (11.11%)	0 (0.00%)
**On a scale from 1 to 5 (with 5 being mostly relevant), when do you think the pregnant woman needs to be informed in the following situation?**							
		**1**	**2**	**3**	**4**	**5**	
When the measurements doesn't have any abnormalities	9	1 (11.11%)	4 (44.44%)	1 (11.11%)	2 (22.22%)	1 (11.11%)	
When the measurements can't be overviewed due to technical problems	9	1 (11.11%)	1 (11.11%)	2 (22.22%)	3 (33.33%)	2 (22.22%)	
When an adequate follow-up isn't possible due to insufficient compliance by the patient	9	0 (0.00%)	1 (11.11%)	1 (11.11%)	4 (44.44%)	3 (33.33%)	
When the measurements can't be overviewed due to problems by the online dashboard	9	1 (11.11%)	0 (0.00%)	1 (11.11%)	5 (55.55%)	2 (22.22%)	
When the privacy of the pregnant women is threated due to problems with the data security	9	1 (11.11%)	2 (22.22%)	0 (0.00%)	5 (55.56%)	1 (11.11%)	
**Of who do you think the pregnant woman needs to receive an overview or feedback about the results … (Multiple answers are correct)**							

		**Autom-atically**	**The gynea-cologist**	**The mid-wife**	**The general practi-tioner**	**The resear-cher**	**No prefer-ence**	**Other**
When there are no abnormal measurements	9	1 (11.11%)	4 (44.44%)	1 (11.11%)	0 (0.00%)	3 (33.33%)	0 (0.00%)	0 (0.00%)
When there are abnormal measurements	9	0 (0.00%)	7 (77.78%)	0 (0.00%)	0 (0.00%)	2 (22.22%)	0 (0.00%)	0 (0.00%)
**Do you think that the partner of the pregnant women needs to be involved in the services remote monitoring offers?**								
		**Yes**				**No**		
	9	2 (22.22%)				7 (77.78%)		
**On a scale from 1 to 5 (with 5 being mostly relevant), how would you involve the partner of the pregnant woman in this program?**								
		**1**	**2**	**3**	**4**	**5**		
By involving the partner in the intake conversation	9	2 (22.22%)	0 (0.00%)	3 (33.33%)	2 (22.22%)	2 (22.22%)		
By giving the partner an overview of the results of the pregnant women on his Smartphone	9	6 (66.67%)	2 (22.22%)	1 (11.11%)	0 (0.00%)	0 (0.00%)		
By informing the partner when measurements are missed	9	4 (44.44%)	1 (11.11%)	3 (33.33%)	0 (0.00%)	1 (11.11%)		
By giving feedback of the results to the partner	9	4 (44.44%)	2 (22.22%)	2 (22.22%)	0 (0.00%)	1 (11.11%)		

9. Privacy

Variable	N	Response frequencies				
**Rate the following questions on a scale from 1 to 5 (with 5 being mostly agree).**						
		**1**	**2**	**3**	**4**	**5**
Would you offer remote monitoring to your patients when their privacy can't guaranteed fully?	9	5 (55.56%)	2 (22.22%)	1 (11.11%)	1 (11.11%)	0 (0.00%)
Do you think that remote monitoring is an threat for the patient data?	9	2 (22.22%)	2 (22.22%)	3 (33.33%)	2 (22.22%)	0 (0.00%)
How import is patient privacy towards the ability to identify gestational complications in an early stage?	9	1 (11.11%)	3 (33.33%)	2 (22.22%)	3 (33.33%)	0 (0.00%)

10. Financial considerations

Variable	N	Response frequencies			
**What is an acceptable price to pay for remote monitoring?**					
		**€10/month**	**€25/month**	**€50/month**	**Other**
Yes	8	6 (75.00%)	1 (12.50%)	1 (12.50%)	0 (0.00%)

**Who has to pay for remote monitoring?**								
		**The patient**	**The gynea-cologist**	**The society**	**The hospital**	**The gynae-cologist & the society**	**The patient & the society**	**The patient, the gynae-cologist and the society**
Yes	8	1 (12.50%)	0 (0.00%)	1 (12.50%)	0 (0.00%)	0 (0.00%)	6 (75.00%)	0 (0.00%)

**Do you think a distinction has to be made in a reimbursement by the insurance between patients with a low risk or a high risk?**			
		**Yes**	**No**
	8	6 (75.00%)	2 (25.00%)

**Do you think that the cost-saving of remote monitoring exceed the additional costs of remote monitoring?**			
		**Yes**	**No**
	8	6 (75.00%)	2 (25.00%)

**On a scale from 1 to 5 (with 5 being mostly relevant), which items are important to implement remote monitoring into daily practice?**						
		**1**	**2**	**3**	**4**	**5**
An additional training (with an certificate)	8	0 (0.00%)	0 (0.00%)	1 (12.50%)	6 (75.00)	1 (12.50%)
Hiring additional staff	8	0 (0.00%)	0 (0.00%)	1 (12.50%)	7 (87.50%)	0 (0.00%)
When an independent service will follow-up the patients and gives us information about the remote monitoring services	8	0 (0.00%)	1 (12.50%)	6 (75.00%)	1 (12.50%)	0 (0.00%)
When this is an activity which only happens during the working hours	8	4 (50.00%)	2 (25.00%)	1 (12.50%)	1 (12.50%)	0 (0.00%)
When additional space will be made into the work planning so no extra workload will be created	8	0 (0.00%)	2 (25.00%)	2 (25.00%)	3 (37.50%)	1 (12.50%)

11. Expansion

Variable	N	Response frequencies				
**Rate the following questions on a scale from 1 to 5 (with 5 being mostly agree).**						
		**1**	**2**	**3**	**4**	**5**
I support the remote monitoring services and recommend is to my patients	8	0 (0.00%)	0 (0.00%)	3 (37.50%)	2 (25.00%)	3 (37.50%)
I would recommend this services to my colleges	8	0 (0.00%)	0 (0.00%)	2 (25.00%)	3 (37.50%)	3 (37.50%)
I would expand this remote monitoring services over all the hospitals in Belgium	8	0 (0.00%)	0 (0.00%)	3 (37.50%)	3 (37.50%)	2 (25.00%)
