# Supplementary material for: Midwives’, Obstetricians’, and Recently Delivered Mothers’ Perceptions of Remote Monitoring for Prenatal Care: Retrospective Survey
Source: J Med Internet Res. 2019 Apr 15;21(4):e10887. doi: 10.2196/10887 (PMC6487343; doi:10.2196/10887)
Supplement: Multimedia Appendix 3 [file jmir_v21i4e10887_app3.pdf]

Questionnaire for Patients

1. Personal information

Variable	N	Response frequencies				
**What was your age at the start of your pregnancy**						
**< 20 years**	**21-25 years**	**26-30 years**	**31-35 years**	**36-40 years**	**> 40 years**	**< 20 years**
0<br>(0.00%)	5<br>(10.64%)	16<br>(34.04%)	21<br>(44.68%)	4<br>(8.51%)	1<br>(2.13%)	0<br>(0.00%)
**How many pregnancies did you have before this project**						
**1**	**2**	**3**	**4**	**≥ 5**		
21<br>(44.68%)	16<br>(34.04%)	8<br>(17.02%)	2<br>(4.26%)	0<br>(0.00%)		
**Did you experience a high blood pressure in a previous pregnancy**						
**Yes**		**No**		**Inapplicable**		
17<br>(36.17%)		10<br>(21.28%)		20<br>(42.55%)		
**What is your highest education level**						
**Lower secondary education**	**Higher secondary education**	**Graduate school**	**University**			
4<br>(8.51%)	12<br>(25.53%)	20<br>(42.55%)	11<br>(23.40%)			
**Did you already had a smartphone?**						
**Yes**		**No**				
45<br>(95.74%)		2<br>(4.26%)				
**Would you buy a smartphone only for telemonitoring during your pregnancy? Or would you buy another type of smartphone if your current device would not be able to be used for telemonitoring**						
Yes, I would buy one. Even without problems during my pregnancy	Yes, I would buy one if I was told that I have a higher risk to develop preeclampsia during my pregnancy	No, I would rather rent a device and pay a premium, even without problems during pregnancy.	No, I would rather rent a device and pay a premium if I was told that I have a higher risk to develop preeclampsia during my pregnancy	No, I would not buy or rent such a device	Yes, I would buy one. Even without problems during pregnancy	
6<br>(12.77%)	10<br>(21.28%)	3<br>(6.38%)	23<br>(48.94%)	5<br>(10.64%)	6<br>(12.77%)	
**At what week of your pregnancy would you start to be followed by telemonitoring?**						
**<10 weeks**	**10-15 weeks**	**15-20 weeks**				
12<br>(25.53%)	15<br>(31.91%)	10<br>(21.28%)				

2. Privacy

Variable	N	Response frequencies				
**Rate the following questions on a scale from 1 to 5 (with 5 being mostly agree).**						
		**1**	**2**	**3**	**4**	**5**
Do you consider the sharing of data (weight, blood pressure and activity) with your doctor as a privacy threat	47	36<br>(76.6%)	5<br>(10.64%)	2<br>(4.26%)	1<br>(2.13%)	3<br>(6.38%)

3. Quality and patient safety

Variable	N	Response frequencies					
**Rate the following questions on a scale from 1 to 5 (with 5 being mostly agree).**							
		**1**	**2**	**3**	**4**	**5**	
This study is important for the follow-up of pregnancies	47	0 (0.00%)	1 (2.13%)	4 (8.51%)	13 (27.66%)	29 (61.70%)	
Telemonitoring gives a feeling of safety	47	0 (0.00%)	1 (2.13%)	7 (14.89%)	15 (31.91%)	24 (51.06%)	
There was problem to perform the measurements at the appropriate time	47	7 (14.89%)	17 (36.17%)	9 (19.15%)	11 (23.40%)	3 (6.38%)	
Performing the measurements was difficult due work	47	33 (70.21%)	10 (21.28%)	0 (0.00%)	1 (2.13%)	3 (6.38%)	
Performing the measurements was difficult due private life (e.g family life)	47	13 (27.66%)	21 (44.68%)	7 14.89%)	3 (6.38%)	3 (6.38%)	
Performing the measurements was difficult due my personality (e.g. scatteredness)	47	21 (44.68)	10 (21.28%)	6 (12.77%)	8 (17.02%)	2 (4.26%)	
Performing the measurements causes difficulty in work	47	36 (76.60%)	6 (12.77%)	1 (2.13%)	1 (2.13%)	3 (6.38%)	
Performing the measurements causes difficulty in private life	47	32 (68.09%)	11 (23.40%)	2 (4.26%)	0 (0.00%)	2 (4.26%)	
There was no problem to perform the measurements at the appropriate time	47	1 (2.13%)	6 (12.77%)	6 (12.77%)	20 (42.55%)	14 (29.79%)	
**Please indicate at the scale below the evaluation of adherence for performing the measurements during your pregnancy (2x/day blood pressure monitoring, 1x/day weight and continue measurement of activity)**							
		**Inapplicable**	**Not consistens at all**	**Often not consistent**	**Occasionally consistent**	**Often consistent**	**Very consistent**
10 – 15 weeks	47	35 (74.47%)	1 (2.13%)	0 (0.00%)	0 (0.00%)	3 (6.38%)	8 (17.02%)
15 – 20 weeks	47	25 (53.19%)	0 (0.00%)	1 (2.13%)	0 (0.00%)	9 (19.15%)	12 (25.53%)
20 – 25 weeks	47	19 (40.43%)	0 (0.00%)	0 (0.00%)	1 (2.13%)	14 (29.79%)	13 (27.66%)
25 – 30 weeks	47	12 (25.53%)	2 (4.26%)	0 (0.00%)	3 (6.38%)	15 (31.91%)	15 (31.91%)
30 – 35 weeks	47	12 (25.53%)	1 (2.13%)	1 (2.13%)	3 (6.38%)	12 (25.53%)	18 (38.30%)
**Rate the following questions on a scale from 1 to 5 (with 5 being mostly agree).**							
		**1**	**2**	**3**	**4**	**5**	
Performing measurements was	47	39 (82.98%)	3 (6.38%)	2 (4.26%)	1 (2.13%)	2 (4.26%)	

difficult due physical discomfort							
Performing measurements was difficult due time related issues	47	21 (44.68%)	9 (19.15%)	10 (21.28%)	1 (2.13%)	6 (12.77%)	
Performing measurements was difficult due device related issues	47	13 (27.66%)	6 (12.77%)	7 (14.89%)	14 (29.79%)	7 (14.89%)	
Performing measurements was difficult because of remembering issues	47	15 (31.91%)	13 (27.66%)	9 (19.15%)	6 (12.77%)	4 (8.51%)	
Performing measurements was difficult because of motivation issues	47	33 (70.21%)	9 (19.15%)	2 (4.26%)	2 (4.26%)	1 (2.13%)	
Performing measurements was difficult because of the confrontation with the results	47	32 (68.09%)	9 (19.15%)	4 (8.51%)	1 (2.13%)	1 (2.13%)	
Performing the measurements was difficult due the disturbing of the measurements	47	37 (78.72%)	7 (14.89%)	2 (4.26%)	0 (0.00%)	1 (2.13%)	
I found the measurements less important during the progression of my pregnancy	47	34 (72.34%)	5 (10.64%)	4 (8.51%)	3 (6.38%)	1 (2.13%)	
There were no difficulties in the performing of the measurements	47	11 (23.40%)	4 (8.51%)	11 (23.40%)	10 (21.28%)	11 (23.40%)	
**Rate the following questions on a scale from 1 to 5 (with 5 being mostly agree).**							
		**1**	**2**	**3**	**4**	**5**	
I found it difficult to use telemonitoring technology	47	29 (61.70%)	13 (27.66%)	3 (6.38%)	1 (2.13%)	1 (2.13%)	
I found it difficult to use telemonitoring technology because I did not always have my smartphone with me	47	32 (68.09%)	9 (19.15%)	5 (10.64%)	1 (2.13%)	0 (0.00%)	
I found it difficult to use telemonitoring technology because the technology was too difficult	47	29 (61.70%)	12 (25.53%)	5 (10.64%)	0 (0.00%)	1 (2.13%)	
I found it difficult to use telemonitoring technology because I do not trust medical smartphone applications	47	40 (85.11%)	6 (12.77%)	1 (2.13%)	0 (0.00%)	0 (0.00%)	
I found it difficult to use telemonitoring technology because	47	32 (68.09%)	8 (17.02%)	5 (10.64%)	1 (2.13%)	1 (2.13%)	

the measurements caused an additional effort and took a lot of time							
**Should every pregnant be followed with telemonitoring?**							
**Yes, independent from pregnancy problems**		**Yes, but only when there are problems in the pregnancy**		**Yes, but only when there were problems in a previous pregnancy**		**No, nobody should be followed with telemonitoring**	
14 (29.79%)		24 (51.06%)		9 (19.15%)		0 (0.00%)	
**Would you consider participating again in such studies (multiple answers are possible)**							
**Yes, independent from a high-risk pregnancy**		**Yes, but only in a high-risk pregnancy**		**Yes, I should also be participating in other studies about telemonitoring in obstetrics**		**No, I will not be participating in such studies**	
24 (51.06%)		15 (31.91%)		14 (29.79%)		0 (0.00%)	

4. Feedback

Within how much time do you want to be contacted about events?'						
**< 3 hours**	**3 – 12 hours**	**12 – 24 hours**	**24 – 48 hours**			
13 (27.66%)	19 (40.43%)	7 (14.89%)	5 (10.64%)			
**Variable**	**N**	**Response frequencies**				
**Rate the following questions on a scale from 1 to 5 (with 5 being mostly agree). When insufficient measurements are performed to provide optimal follow-up, the healthcare provider contacts the patients by:**						
		**1**	**2**	**3**	**4**	**5**
SMS	47	7 (14.89%)	3 (6.38%)	7 (14.89%)	14 (29.79%)	16 (34.04%)
Telephone	47	3 (6.38%)	4 (8.51%)	9 (19.15%)	10 (21.28%)	21 (44.68%)
Mail	47	12 (25.53%)	7 (14.89%)	14 (29.79%)	6 (12.77%)	8 (17.02%)
**Rate the following questions on a scale from 1 to 5 (with 5 being mostly agree). The appropriate way for the patient to contact the healthcare provider for device related problems**						
		**1**	**2**	**3**	**4**	**5**
SMS	47	10 (21.28%)	3 (6.38%)	14 (29.79%)	12 (25.53%)	8 (17.02%)
Telephone	47	2 (4.26%)	0 (0.00%)	5 (10.64%)	10 (21.28%)	30 (63.83%)
Mail	47	8 (17.02%)	3 (6.38%)	9 (19.15%)	13 (27.66%)	14 (29.79%)
SMS	47	10 (21.28%)	3 (6.38%)	14 (29.79%)	12 (25.53%)	8 (17.02%)
**Rate the following questions on a scale from 1 to 5 (with 5 being mostly agree). The appropriate way for the healthcare provider to contact the patient for device related problems:**						
		**1**	**2**	**3**	**4**	**5**
SMS	47	11 (23.40%)	3 (6.38%)	9 (19.15%)	12 (25.53%)	12 (25.53%)
Telephone	47	2 (4.26%)	1 (2.13%)	5 (10.64%)	10 (21.28%)	29 (61.70%)
Mail	47	9 (19.15%)	8 (17.02%)	10 (21.28%)	9 (19.15%)	11 (23.40%)
**Rate the following questions on a scale from 1 to 5 (with 5 being mostly agree). When the measured values are abnormal (e.a. high blood pressure) and the healthcare provider wants to inform the patient about this to provide customized advice, they can contact the patient by:**						
		**1**	**2**	**3**	**4**	**5**
SMS	47	12 (25.53%)	5 (10.64%)	6 (12.77%)	11 (23.40%)	13 (27.66%)
Telephone	47	2 (4.26%)	1 (2.13%)	2 (4.26%)	6 (12.77%)	36 (76.60%)
Mail	47	19 (40.43%)	5 (10.64%)	7 (14.89%)	11 (23.40%)	5 (10.64%)
At consultation	47	5 (10.64%)	1 (2.13%)	8 (17.02%)	11 (23.40%)	22 (46.81%)
**Please indicate the preferable way and schedule to receive feedback when the measured values are normal. (multiple answers are possible)**						
		**SMS**	**Telephone**	**Mail**	**At consultation**	**No overview**
An overview of the results at daily basis	47	14 (29.79%)	10 (21.28%)	23 (48.94%	10 (21.28%)	10 (21.28%)

An overview of the results at weekly basis	47	13 (27.66%)	10 (21.28%)	32 (68.09%)	15 (31.91%)	4 (8.51%)
An overview of the results at monthly basis	47	11 (23.40%)	11 (23.40%)	33 (70.21%)	23 (48.94)	2 (4.26%)
**Who would be the favorable persons to contact you following an event.**						
**Obstetrician**	**Midwife**	**General practitioner**	**Researcher**	**No preference**		
40 (85.11%)	25 (53.19%)	9 (19.15%)	11 (23.40%)	6 (12.77%)		
**Does your partner needs to be more involved in telemonitoring**						
**Yes**		**No**				
13 (27.66%)		34 (72.34%)				
**Rate the following questions on a scale from 1 to 5 (with 5 being mostly agree). The appropriate way for your partner to be more involved in telemonitoring**						
		**1**	**2**	**3**	**4**	**5**
By involving the partner in the intake conversation	47	15 (31.91%)	4 (8.51%)	7 (14.89%)	7 (14.89%)	14 (29.79%)
By giving the partner an overview of the results of the pregnant women on his Smartphone	47	20 (42.55%)	7 (14.89%)	8 (17.02%)	7 (14.89%)	5 (10.64%)
By informing the partner when measurements are missed	47	18 (38.30%)	11 (23.40%)	8 (17.02%)	7 (14.89%)	3 (6.38%)
By giving feedback of the results to the partner	47	15 (31.91%)	7 (14.89%)	11 (23.40%)	7 (14.89%)	7 (14.89%)

5. Financial information

**Are you willing to pay 10 euro per month for the telemonitoring service**											
**Yes**		**No**									
12 (52.17%)		11 (47.83%)									
**Are you willing to pay 25 euro per month for the telemonitoring service**											
**Yes**		**No**									
4 (40.00%)		6 (60.00%)									
**Are you willing to pay 50 euro per month for the telemonitoring service**											
**Yes**		**No**									
3 (23.08%)		10 (76.92%)									
**If you have negatively answered the previous question, how much are you willing to pay for the telemonitoring service?**											
**€0**		**€10**		**€15**		**€25**		**€30**			
14		1		4		1		1			
**Variable**		**N**	**Response frequencies**								
			**1**	**2**	**3**	**4**	**5**				
The telemonitoring service should be offered by the hospital.		46	2 (4.35%)	0 (0.00%)	7 (15.22%)	12 (26.09%)	25 (54.35%)				
The telemonitoring service should be offered by mutualities.		46	4 (8.70%)	3 (6.52%)	7 (15.22%)	9 (19.57%)	23 (50.00%)				
The telemonitoring service should be offered by social security.		46	5 (10.87%)	5 (10.87%)	15 (32.61%)	8 (17.39%)	13 (28.26%)				
I do not care who offers the telemonitoring service.		46	7 (15.22%)	5 (10.87%)	6 (13.04%)	5 (10.87%)	23 (50.00%)				
**Please indicate the following questions with a score from 0 – 10.**											
		**1**	**2**	**3**	**4**	**5**	**6**	**7**	**8**	**9**	**10**
How satisfied are you about the telemonitoring service	46	0 (0.00%)	0 (0.00%)	1 (2.17%)	1 (2.17%)	1 (2.17%)	3 (6.52%)	5 (10.87%)	15 (32.61%)	12 (26.09%)	8 (17.39%)
How satisfied are you about the telemonitoring devices	46	1 (2.17%)	0 (0.00%)	1 (2.17%)	4 (8.70%)	2 (4.35%)	6 (13.04%)	11 (23.91%)	9 (19.57%)	7 (15.22%)	5 (10.87%)
Would you recommend telemonitoring to family or friends	46	1 (2.17%)	0 (0.00%)	1 (2.17%)	0 (0.00%)	2 (4.35%)	3 (6.52%)	7 (15.22%)	10 (21.74%)	13 (28.26%)	9 (19.57%)
